# Mitochondrial neurogastrointestinal encephalomyopathy: approaches to diagnosis and treatment

**DOI:** 10.20517/jtgg.2020.08

**Published:** 2020-03-30

**Authors:** Bridget E. Bax

**Affiliations:** Institute of Molecular and Clinical Sciences, St. George’s University of London, London, SW17 ORE, UK

**Keywords:** Mitochondrial neurogastrointestinal encephalomyopathy, MNGIE, thymidine phosphorylase, *TYMP*, mitochondrial DNA, mitochondrial disease

## Abstract

Mitochondrial neurogastrointestinal encephalomyopathy (MNGIE) is an ultra-rare disease caused by mutations in *TYMP,* the gene encoding for the enzyme thymidine phosphorylase. The resulting enzyme deficiency leads to a systemic accumulation of thymidine and 2’-deoxyuridine and ultimately mitochondrial failure due to a progressive acquisition of secondary mitochondrial DNA (mtDNA) mutations and mtDNA depletion. MNGIE is characterised by gastrointestinal dysmotility, cachexia, peripheral neuropathy, ophthalmoplegia, ptosis and leukoencephalopathy. The disease is progressively degenerative and leads to death at an average age of 37.6 years. Patients invariably encounter misdiagnoses, diagnostic delays, and non-specific clinical management. Despite its rarity, MNGIE has invoked much interest in the development of therapeutic strategies, mainly because it is one of the few mitochondrial disorders where the molecular abnormality is metabolically and physically accessible to manipulation. This review provides a resume of the current diagnosis and treatment approaches and aims to increase the clinical awareness of MNGIE and thereby facilitate early diagnosis and timely access to treatments, before the development of untreatable and irreversible organ damage.

## Introduction

Mitochondrial neurogastrointestinal encephalomyopathy (MNGIE; Online Mendelian inheritance in Man #603041, Genome Database accession #9835128) is a fatal inherited metabolic disorder caused by mutations in a nuclear gene impacting on the replication and expression of the mitochondrial genome^[1,2]^. The disorder was first described by Okamura *et al.^[3]^* in 1976 as congenital oculoskeletal myopathy. Patients with ocular, neurological, skeletal and gastrointestinal involvement were subsequently described, with use of the following nomenclatures: mitochondrial myopathy with sensorimotor polyneuropathy, ophthalmoplegia and pseudo-obstruction (MEPOP); mitochondrial neurogastrointestinal encephalopathy syndrome; myoneurogastrointestinal encephalopathy syndrome; chronic intestinal pseudo-obstruction with myopathy and ophthalmoplegia; polyneuropathy, ophthalmoplegia, leukoencephalopathy and intestinal pseudoobstruction (POLIP); oculogastrointestinal encephalopathy syndrome; oculogastrointestinal muscular dystrophy (OGIDM); and thymidine phosphorylase deficiency^[4–8]^. Hirano *et al*.^[9]^ in 1994 proposed the current nomenclature, MNGIE, as it precisely describes the key features of this mitochondrial disorder. The aetiology of MNGIE was later attributed to a deficiency in the enzyme thymidine phosphorylase (Enzyme Commission 2.4.2.4)^[1]^.

**Figure 1:**
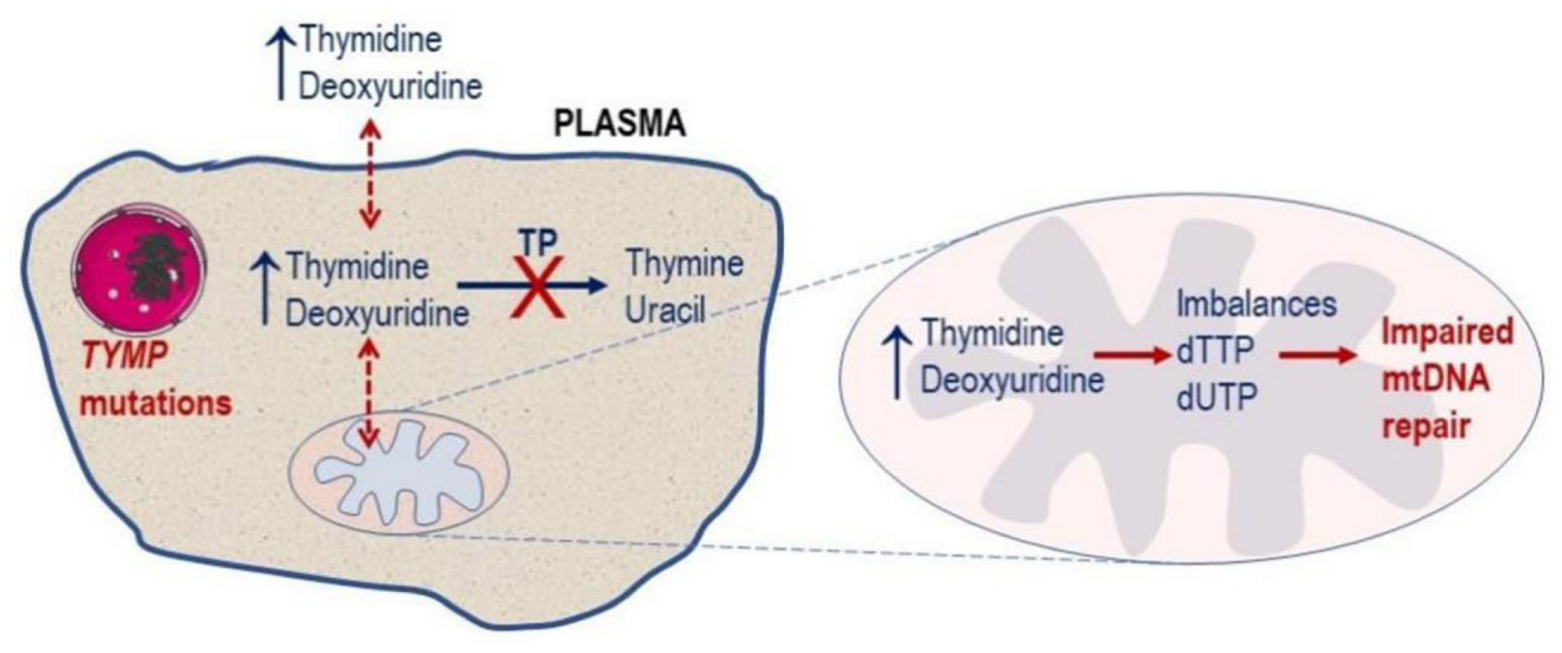
Molecular defect in MNGIE. Pathogenic mutations in the nuclear *TYMP* gene leads to a deficiency in TP activity, resulting in a plasma and cytosolic accumulation of the enzyme’s substrates, thymidine and 2’-deoxyuridine. This leads to imbalances in the mitochondrial deoxyribonucleotide pools (dTTP and dUTP) and ultimately impaired mtDNA repair. TP: thymidine phosphorylase; MNGIE: mitochondrial neurogastrointestinal encephalomyopathy; mtDNA: mitochondrial DNA

MNGIE is an autosomal recessive disease caused by loss of function mutations in *TYMP* (previously known as *ECGF_1_*), a nuclear gene located on chromosome 22 which encodes for thymidine phosphorylase. The protein in the past has also been referred to as gliostatin and platelet derived-endothelial cell growth factor due to its growth inhibitory factor and angiogenic factor activities, respectively. Thymidine phosphorylase catalyses the reversible phosphorylation of the pyrimidine deoxyribonucleosides, thymidine (deoxythymidine) and 2’-deoxyuridine to 2-deoxyribose 1-phosphate and their respective bases, thymine and uracil^[1]^. Pathogenic mutations in *TYMP* cause a complete or partial deficiency in enzyme activity, which leads to a measurable systemic accumulation of thymidine and 2’-deoxyuridine and, consequently, elevated intracellular concentrations of their corresponding triphosphates. These perturbations affect the physiological equilibrium of the four deoxyribonucleotides within the mitochondria, thereby interfering with the normal replication of mitochondrial DNA (mtDNA), leading to multiple deletions, somatic point mutations and depletion of mtDNA, and ultimately mitochondrial failure^[1,9–14]^[Figure 1]. mtDNA encodes for polypeptides, transfer RNA and ribosomal RNA required for the synthesis of enzymes involved in oxidative phosphorylation. The consequent failure of cellular energy production is believed to directly cause the cardinal central clinical manifestations through damage to the nervous and muscular systems.

MNGIE is a progressive, multi-system disease, with a characteristic, although by no means universal, clinical presentation with gastrointestinal symptoms, including early satiety, dysphagia, nausea, chronic abdominal pain and diarrhoea leading to weight loss. These symptoms are secondary to alimentary dysmotility caused by degeneration of the alimentary autonomic nervous system. Patients generally have a thin body habitus with reduced muscle mass and cachexia is common. Episodes of frank intestinal pseudo-obstruction may occur and some patients develop a hepatopathy with liver steatosis and cirrhosis. Progressive external ophthalmoplegia and peripheral sensorimotor polyneuropathy are invariable, with the latter affecting the lower limbs initially. On magnetic resonance imaging (MRI), there is, in most cases, diffuse increased T_2_ signal in the cerebral hemispheres but this is usually asymptomatic^[2,9,10]^. Patient survival is generally related to the degree of gastrointestinal involvement, with patients dying at an average age of 37.6 years as a result of cachexia, peritonitis, oesophageal bleeding, intestinal rupture or aspiration pneumonia^[10,15,16]^.

MNGIE is an ultra-rare disorder with an estimated European prevalence of 1 in 1,000,000 (Orphanet report, 2019), although this figure is entirely dependent on evidence reported in the literature and therefore cannot be considered absolutely correct^[17]^. The disease is reported to be widely distributed amongst an ethnically diverse population including Hispanics, Africans, Americans, Western Europeans, Chinese, Indians, Ashkenazi Jewish, Middle Eastern and Canadians^[2,18–25]^. Due to the rarity of MNGIE and its complex multisystem clinical picture, patients typically undergo a number of different specialty referrals over many years before obtaining a correct diagnosis^[26,27]^. Despite its rarity, MNGIE has invoked much interest in the field of mitochondrial diseases, mainly because it is one of the few mitochondrial disorders where the molecular abnormality is metabolically and physically accessible to manipulation.

The aim of this article is to provide a review of the current approaches to the diagnosis of MNGIE and chronicle the standard and investigational therapeutic strategies that have been applied to treat this disorder.

## Diagnosis of MNGIE

### Disease onset

The average age of MNGIE disease onset is generally within the first and second decades of life, with an average of 18.5 years of age, although cases have been reported where onset was as early as five months of age and as late as the fifth decade^[16,19,28,29]^. The relatively late onset for a condition present at birth is thought to be due to the progressive accumulation of mtDNA defects, with the disease becoming apparent once the number of affected mitochondria reaches a critical threshold level. In the largest cohort reported to date, for the majority of patients, the first non-specific symptoms manifested during childhood, therefore challenging the accuracy of the reported age of onset. Non-specific symptoms included gastrointestinal in approximately 50% of the patients, ocular in 20%, and the majority of the remaining having neuropathic and/or myopathic symptoms at onset^[16]^.

### Misdiagnoses and diagnosis delays

The complex clinical presentation of MNGIE is often difficult for the non-specialist healthcare professional to interpret, and particularly as some of the clinical hallmark features of the disorder may be absent early on in the disease course. Consequently, patients often experience misdiagnoses and diagnostic delays^[16]^. Incorrect initial diagnoses that have been reported include anorexia nervosa and other gastrointestinal disorders such as Crohn’s disease and inflammatory bowel disease, esophagitis and/or gastritis, Whipple disease, chronic intestinal pseudo-obstruction, coeliac disease, irritable bowel syndrome and superior mesenteric artery syndrome^[15,16,30–33]^. The speed at which the neuropathic symptoms develop has resulted in misdiagnoses of chronic inflammatory demyelinating polyneuropathy or Charcot-Marie-Tooth disease in some patients^[34–36^. The disorder has also been misdiagnosed as other mitochondrial DNA depletion syndromes such as *POLG* or *RRM_2_B* mutations or other mitochondrial diseases, including Kearns-Sayre syndrome and chronic progressive external ophthalmoplegia^[16,37,38]^. Many patients undergo invasive diagnostic procedures, for example exploratory surgery, and sometimes repeatedly, due to episodes of pseudo-obstruction, or unnecessary therapies, for example intravenous immunoglobulin^[39–41]^. As with many other rare diseases, diagnostic delays are not uncommon for patients with MNGIE, with reported delays of between 5 and 10 years^[26,27]^. Due to the progressive nature of the disease, a late diagnosis is often associated with a poor response to therapy and subsequent poor prognosis^[42,43^.

### Diagnostic methods

#### Clinical investigations

Patients typically undergo referral to a number of clinical specialties before obtaining a correct diagnosis through biochemical testing and *TYMP* sequencing. Clinical signs which raise a suspicion of MNGIE are gastrointestinal dysmotility, cachexia, peripheral neuropathy, ptosis and external ophthalmoplegia^[2,3,16]^. To understand the basis of the of the presenting symptoms, patients invariably undergo a number of different diagnostic procedures.

To evaluate the gastrointestinal symptoms, a range of diagnostic modalities are used and include abdominal ultrasound, abdominal imaging (X-ray and computed tomography), upper gastrointestinal tract contrast radiography, esophagogastroduodenoscopy, sigmoidoscopy, colonoscopy, liquid phase scintigraphy, antroduodenal manometry, biopsy and histopathological examination^[44–46]^. These assessments often reveal hepatomegaly, hepatosteatosis, dilatation and thickening of the stomach and small intestine, jejunum diverticulosis, reduced gastroduodenal motility and transit, decreased lower oesophageal sphincter pressure, a low amplitude of oesophageal contractions, gastroparesis, reflux oesophagitis, panagastritis, non-specific chronic inflammation in the small intestine and foamy cells in the intestinal wall^[21,23,30,47–55]^.

Diagnostic techniques used to evaluate the neuromuscular and neurological symptoms include electromyography to assess the presence, duration and severity of myopathy; nerve conduction velocity to assess the extent of the peripheral nervous system involvement; and brain MRI to confirm leukoencephalopathy. The peripheral neuropathy symptoms manifest as paraesthesia with a stockingglove distribution and profound limb weakness and muscle atrophy, prominently affecting the lower extremities^[16]^. Unilateral or bilateral foot drop, as well as clawed hands, may occur. Diagnostic assessments show decreased motor and sensory nerve conduction velocities and prolonged F-wave, indicating severe demyelinating and axonal sensorimotor polyneuropathy^[2]^. The segmental demyelination is thought to be a result of an uneven distribution of mtDNA abnormalities along the nerve^[56]^ Asymptomatic leukoencephalopathy is a hallmark of MNGIE and its presence in combination with the gastrointestinal and neurological symptoms significantly narrows the diagnosis to MNGIE^[16]^. In the majority of patients, the leukoencephalopathy is initially patchy but becomes progressively more diffuse, appearing as hypointense on T1- and hyperintense on T2-weighted images and in fluid-attenuated inversion recovery and fast spin echo T2 sequences^[16,43,57,58]^.

Ophthalmological assessments, including visual evoked potential and neuro-ophthalmologic evaluations, have revealed prolonged P100 latency and retinitis, respectively^[29,49]^. Audiology investigations have confirmed unilateral or bilateral sensorineural hearing impairment in some patients^[29,43,59,60]^.

Occasional cardiac complications have been diagnosed in some patients, including abnormal ECG, with individuals displaying left ventricular hypertrophy and bundle branch block, a prolonged QT interval, cardiac arrest and supraventricular tachycardia, mitral valve prolapse and systolic heart murmurs, cardiomyopathy and endocarditis^[2,16,22,47,61]^.

#### Laboratory investigations

Although confirming a diagnosis of MNGIE is usually very straightforward, diagnostic testing is not routinely performed by the majority of chemical pathology laboratories. A genetic confirmation of MNGIE is mandatory. Testing for MNGIE is accomplished by the measurement of plasma and urine thymidine and 2’-deoxyuridine concentrations, the measurement of thymidine phosphorylase activity and *TYMP* sequencing.

Thymidine and 2’-deoxyuridine measurements are performed using high-performance liquid chromatography with ultraviolet spectrophotometric (HPLC-UV) or tandem mass spectrometric detection^[62–64]^. In contrast to healthy unaffected individuals who have undetectable plasma levels of these metabolites (< 0.05 μmol/L), patients with MNGIE have markedly elevated plasma concentrations of thymidine (> 3 μmol/L) and 2’-deoxyuridine (> 5 μmol/L)^[12,13]^. The urinary excretion of thymidine and 2’-deoxyuridine is also increased in patients but attention should be paid to the possibility of metabolite catabolism by contaminating bacteria^[11]^. Preservatives used for chemical urinalysis specimens, such tartaric acid, boric acid, perchloric acid or thymol, should be included in collections that are not analysed immediately.

Thymidine phosphorylase activity is measured either spectrophotometrically or by HPLC-UV by the endpoint determination of the thymine formed after incubation of buffy coat homogenates in the presence of an excess of the enzyme’s substrate, thymidine^[65]^. The assay of enzyme activity is generally required to complement the measurement of plasma metabolite concentrations, or following the identification of novel variants of the *TYMP* gene, or when clinics do not have access to sequencing of *TYMP*. Thymidine phosphorylase activity is severely reduced in the leukocytes of patients with MNGIE, showing either no activity or activities less than 10% (0-46 nmol thymidine formed/hour/mg protein) of healthy unaffected controls (253-1000 nmol thymidine formed/hour/mg protein)^[11,13]^. Heterozygous carriers of *TYMP* mutations have approximately 35% of residual thymidine phosphorylase activity and have undetectable concentrations of plasma deoxyribonucleosides^[66]^.

The benchmark for the diagnosis of MNGIE is the identification of homozygous or compound heterozygous allelic pathogenic *TYMP* variants, which are detected by Sanger or next generation gene sequencing, mitochondrial disease gene panels or whole exosome sequencing. The Human Gene Mutation Database (HGMD Professional 2019.4, accessed January 2020) reports 97 different mutations which have been mapped to exonic or intronic regions, with some identified as benign and other as pathogenic variants^[67]^. These mutations include: 59 missense/nonsense, 14 splice site mutations, 13 small deletions, 7 small insertions, 2 small indels, 1 gross deletion and 1 gross insertion^[68]^. These mutations have been mapped to either exonic or intronic regions, with some identified as benign and the others as pathogenic variants. In the event of a non-identified mutation or variant of uncertain significance being detected, metabolite and thymidine phosphorylase activity testing should be performed to confirm or exclude a diagnosis of MNGIE^[10]^.

Few patients have presented with endocrine and other metabolic dysfunctions, including diabetes, elevations in amylase, glucose intolerance and hypergonadotropic hypogonadism but these are non-specific and largely do not contribute to the diagnosis of MNGIE^[16,21,69]^.

Histological and biochemical studies on skeletal muscle biopsies may reveal abnormalities of a mitochondrial myopathy, including ragged-red fibres, cytochrome c oxidase-deficient fibres, ultra-structurally abnormal mitochondria, mtDNA deletions, depletions or point mutations and decreased single or multiple electron transport chain enzyme activities^[9,45,70,71]^. The absence of mitochondrial abnormalities in skeletal muscle should not be used to preclude a diagnosis of MNGIE as there are reports of patients diagnosed with MNGIE without muscle mitochondrial pathologies^[59,72]^.

## Treatment Options for Patients with MNGIE

### Symptomatic therapies

Currently, there are no specific treatments for patients with MNGIE, whose efficacy has been confirmed in regulatory approved clinical trials. Treatment requires a coordinated approach among multiple specialists, including gastroenterologists, neurologists, pain management teams, nutritionists and physiotherapists, audiologists and ophthalmologists to manage the specific complications this multisystem disease presents.

The gastrointestinal symptoms vary from patient to patient and may include dysmotility, abdominal pain, diarrhoea, nausea, vomiting, premature satiety, borborygmic and dysphagia. Symptoms are treated with bowel motility stimulant drugs, analgesics, anti-emetics, dietary modification and antibiotics, the latter for intestinal bacterial overgrowth, a complication of dysmotility^[45,50]^. For patients suffering from epigastric pain, splanchnic nerve blockade or coeliac plexus blockade with bupivacaine can be employed to reduce pain^[68,73,74]^. Post-prandial emesis and nausea may be controlled through the administration of metoclopramide, domperidone, amitriptyline or bisacodyl^[41,74]^ Gastro-oesophageal reflux related to gastroparesis can be controlled by prokinetic agents^[41]^. Diverticula, secondary to the severe gut dysmotility, may lead to complications of gut perforation and require emergency abdominal surgery^[75]^. Malnutrition is a critical issue for the majority of patients and, although enteral or parenteral nutritional support is often administered, this has not been shown to alter the disease trajectory^[76-78]^. Nasogastric nutrition is poorly tolerated by some patients due to the gastrointestinal dysmotility and long-term total parenteral nutrition use has been reported to be associated with hepatic steatosis and elevated transaminases^[16,47,79]^. The administration of total parenteral nutrition should be carefully considered, as the lipid components are metabolised by the mitochondrion, the site of the primary pathology in MNGIE^[77]^. Portal hypertension, complicated by ascites and oesophageal varices, may also develop and these are treated as in other conditions^[48]^.

The neurological symptoms also vary from case to case, and may include paraesthesia, numbness, pain in the limbs, symmetric and distal muscle weakness, hearing loss and ocular symptoms. Neuropathic pain is treated with centrally acting agents such as amitriptyline, gabapentin and pregabalin^[41,79]^. Physiotherapy and occupational therapy may be required, according to the needs of the patient^[80]^. Ocular symptoms may be treated with corrective lenses and surgery for strabismus or ptosis. Cochlear implants or auditory aids may assist patients with sensorineural hearing loss^[60,81]^. For patients with psychiatric symptoms, antidepressants and antipsychotics may provide benefit.

Medications that interfere with mitochondrial function should be avoided, for example, valproate, phenytoin, chloramphenicol, linezolid, aminoglycosides, volatile anaesthetics and tetracycline. Hepatically metabolised drugs should be administered with care or contraindicated depending on the patient’s liver function^[46]^. Genetic counselling should be offered to individuals diagnosed with MNGIE and their families.

### Investigational causative therapies

MNGIE is relentlessly progressive and degenerative, with an almost universally fatal outcome and there is thus a critical requirement to develop treatments that will provide benefit. Early therapeutic intervention to normalise the metabolic derangement is essential to prevent non-reversible organ damage. Fortunately, the past two decades has seen some exciting advancements in the development of a number of investigational therapeutic strategies for the treatment of MNGIE. The therapeutic strategy common to all the approaches investigated to date is to reduce or eliminate the pathological concentrations of thymidine and 2’-deoxyuridine with the aim of ameliorating the intracellular deoxyribonucleoside imbalances to prevent further mtDNA damage, and ultimately translate the metabolic correction into clinical stabilization or improvement. Currently, there are two experimental treatment strategies. The first is the direct removal of the elevated deoxyribonucleosides and includes haemodialysis and continuous ambulatory peritoneal dialysis (CAPD)^[11,63,74]^. The second strategy is the introduction of the deficient enzyme, thymidine phosphorylase, and includes platelet infusions^[26]^, allogeneic haematopoietic stem cell transplantation (AHSCT)^[46,82,83]^, erythrocyte encapsulated thymidine phosphorylase (EE-TP)^[77,84]^ and orthotopic liver transplant (OLT)^[85]^. These are individually discussed below.

#### Haemodialysis

Haemodialysis was the first treatment proposed for MNGIE and the first approach employed to remove the excess concentrations of metabolites from the circulation. Using this procedure, blood is removed from the patient and dialysed using a semi-permeable membrane to deplete the plasma of thymidine and 2’-deoxyuridine, before being returned back to the patient. The study of Spinazzola *et al*.^[11]^ demonstrated a 50% reduction in the plasma metabolite levels in two patients. However, by 19 h post-dialysis, the metabolite levels returned to pre-treatment concentration. The procedure was subsequently used to treat three further patients between 2 and 19 months^[63,86,74]^. In one patient, there were longterm reductions in the urinary excretion of thymidine and in a second patient there was a reported a reduction in the frequency of vomiting to once every 2-3 days, as opposed to after every meal^[63,74]^. The third patient demonstrated transient reductions in plasma and urine metabolite concentrations, with the metabolites returning to baseline within 24 h of stopping the procedure. Disease progression was also reported in this patient, as demonstrated by worsening of the score for the Ataxia Assessment scale, and declines in the Montreal Cognitive Assessment score and nerve conduction measures^[86]^. For all three cases, 3-4 haemodialysis procedures per week were required to maintain a sustained reduction in deoxyribonucleosides. Although no safety issues were reported in these studies, potential safety issues associated with this procedure include hypotension, fluid over-load and infections from repeated venous access. In addition, dialysis can be a burdensome and invasive treatment, and may reduce the patient’s quality of life.

#### CAPD

**Figure 2:**
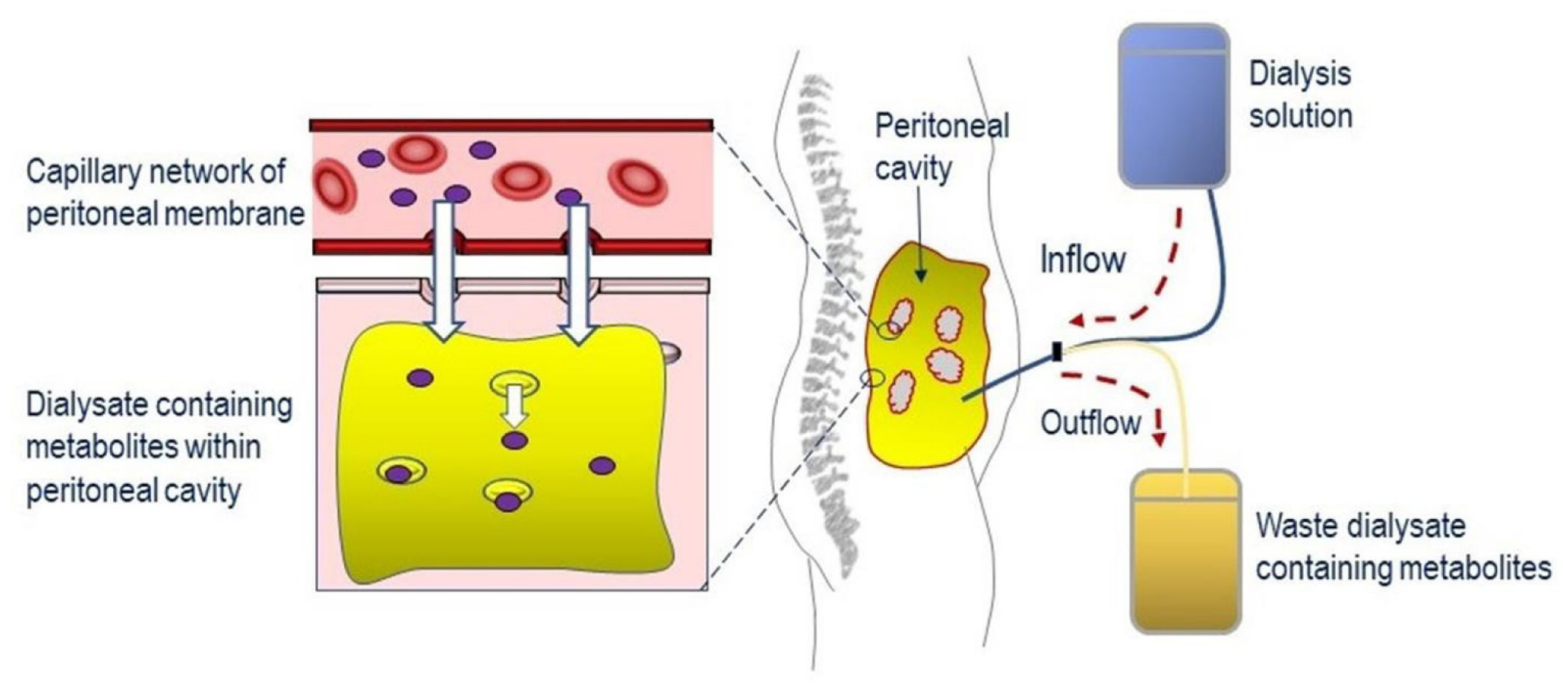
Reduction of systemic concentrations of thymidine and 2’-deoxyuridine through the application of continuous ambulatory peritoneal dialysis. Dialysis solution is infused into the peritoneal cavity and left to dwell for several hours, during which time metabolites (thymidine and 2’-deoxyuridine) pass from the capillary network in the peritoneal membrane into the dialysis solution. The dialysate containing the metabolites is then drained to waste

CAPD involves the filling of the peritoneal cavity with a dialysis solution to encourage the diffusion of thymidine and 2’-deoxyuridine from the blood passing through the capillary network within the peritoneal membrane, and then after several hours of exchange, draining the dialysate containing the metabolites from the peritoneal cavity to waste [Figure 2]. This approach has been reported for the treatment of four single cases, with patients receiving exchanges every 4-8 h, over periods of 22-36 months^[74,87–89]^. Although CAPD had no reported effects on the plasma biochemical imbalances, in one study, it was shown to remove approximately 100 μmol per day of thymidine and 2’-deoxyuridine from the peritoneal cavity ^[74,87]^. Clinical improvements were reported in all four patients and included reductions in vomiting, nausea and epigastric pain; no longer requiring parenteral nutrition; improvements in appetite; increases in body weight (ranging between 3.5 and 13 kg); slight improvement in sensorimotor polyneuropathy and sensory ataxia; and a resolution of the numbness in peripheries. Regained abilities to perform normal daily activities were also reported, for example climb stairs, ambulant without support and improved walking distances^[74,87,88]^. Of interest, many of the clinical improvements reported were gastrointestinal in nature, and CAPD may therefore offer an important approach to targeting the gastrointestinal symptoms of MNGIE. In two cases, the gastrointestinal manifestations reappeared if CAPD was missed or discontinued^[74,88]^. A standardised approach should be implicated in recording body weight gain as the reported increases could be a consequence of fluid retention.

Safety issues associated with the administration of CAPD were catheter infections, mild peritonitis, dialysis fluid leakage and shoulder pain^[74,89]^. Other safety factors associated with this procedure, but were not reported in these four cases, are CAPD-associated peritonitis with or without bowel perforation^[90,91]^. Encapsulating peritoneal sclerosis, which can occur in patients who receive long-term peritoneal dialysis, is also a risk^[92]^. A sclerosed peritoneum may restrict the movement of the bowel, leading to blockages, pain, nausea, vomiting and weight loss, and this is an important consideration, particularly as these symptoms are very similar to those of MNGIE. Another issue to consider is the glucose load associated with the dialysis solutions, particularly as some patients with MNGIE have pancreatic insufficiency. Other concerns associated with CAPD include intestinal perforation during the catheterisation procedure and noncompliance due to patient fatigue, social reasons and occupation.

#### Platelet infusions

Platelets contain high activities of thymidine phosphorylase and the infusion of donor cells into patients with MNGIE was the first method employed to replace the deficient thymidine phosphorylase^[93]^. The rationale of the approach is based on the diffusion of plasma thymidine and 2’-deoxyuridine across the membrane of donor platelets via nucleoside transports into the cells where they are metabolised by the cytosolic-based thymidine phosphorylase into their normal products, thymine and uracil, respectively. Four cases are reported in the literature using this procedure^[87,93,94]^. Reductions in plasma deoxyribonucleoside concentrations, equating to 50%-70% of baseline concentrations, were reported in one patient who received three platelet infusions on Days 0, 4 and 7, with thymidine phosphorylase activity peaking 24 h after each infusion. However, these reductions were transient, with the metabolite concentrations returning to baseline before the administration of the subsequent infusion^[93]^. A second patient showed a total elimination of the plasma metabolites after a single infusion, but again the levels started to increase within a few days. Urinary excretion of metabolites decreased in both patients, with one patient virtually stopping the excretion of nucleosides for 15 days^[93]^. Clinical improvement was reported for one patient, who, four days after receiving the last platelet infusion, was able to walk alone and electrodiagnostic studies showed the disappearance of peroneal and tibial motor nerve conduction block/temporal dispersion between distal and proximal stimulation sites^[87]^. To maintain adequate metabolite reductions, frequent platelet infusions are required, but this can potentially be associated with a number of safety issues, including transfusion-related acute lung injury, allergic reactions, infections and transfusion-associated graft versus host _disease_^[95]^.

#### EE-TP

**Figure 3:**
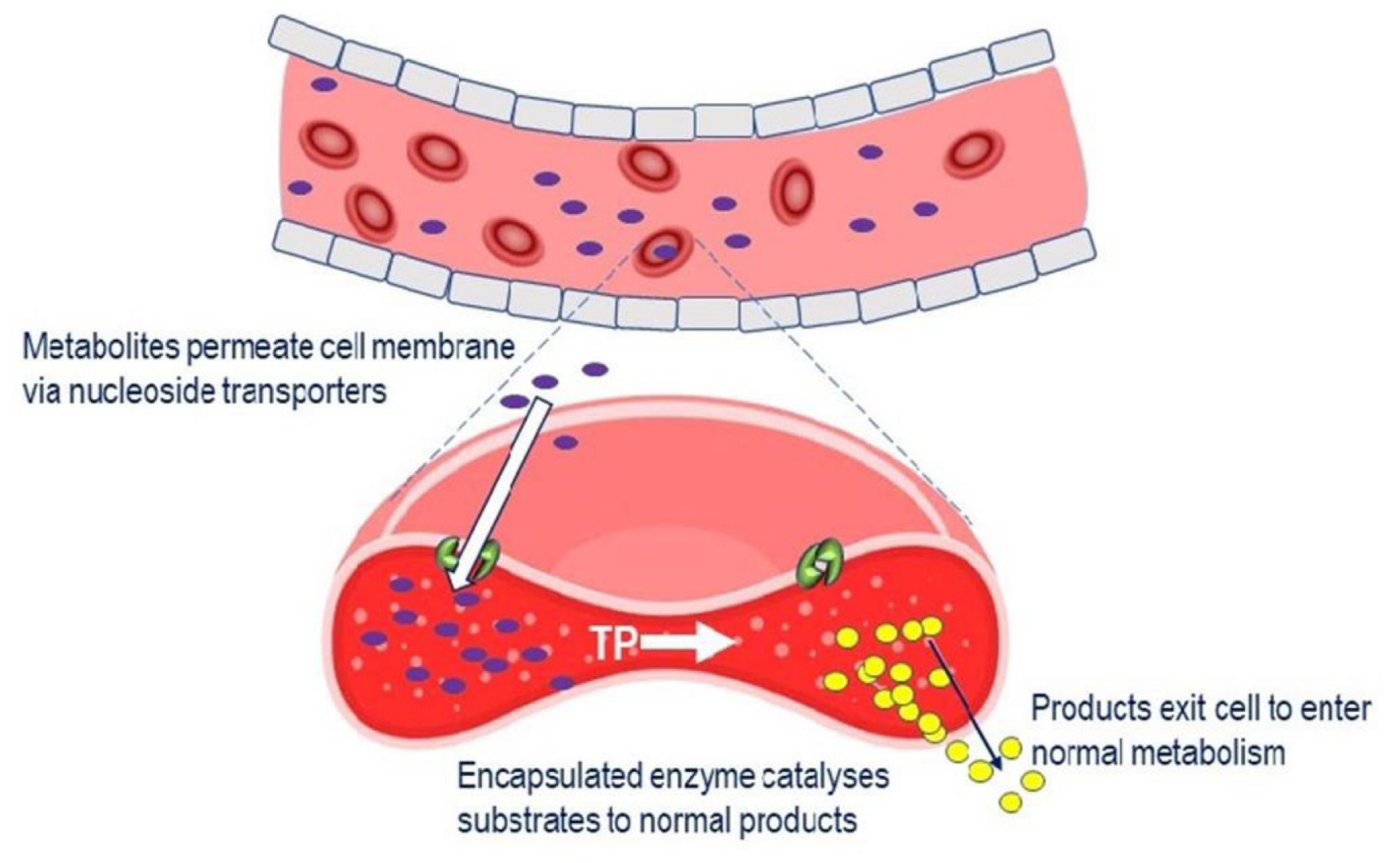
Mechanism of EE-TP action. Plasma thymidine and 2’-deoxyuridine enter the erythrocyte via nucleoside transports located in the cell membrane, where the encapsulated TP catalyses their metabolism to thymine and uracil. The products then exit the cell into the blood plasma where they enter the normal metabolic pathways. TP: thymidine phosphorylase; EE-TP: erythrocyte encapsulated thymidine phosphorylase

Treatment with EE-TP is an alternative approach to enzyme replacement therapy and aims to correct the fundamental lesion in MNGIE by replacement of the deficient enzyme, thymidine phosphorylase, by encapsulating it within the patient’s own (autologous) erythrocytes *ex vitro,* and returning the cells back to the patient. The rationale for the development of EE-TP is the same as for platelet infusions, i.e., thymidine and 2’-deoxyuridine diffusing from the blood plasma across the erythrocyte membrane via nucleoside transporters^[96]^. Once in the cytoplasm, the encapsulated thymidine phosphorylase catalyses the metabolism of the metabolites to the normal products, which are then free to diffuse out of the cell into the blood plasma where they will be further metabolised as normal [Figure 3]. Regular intravenous administrations of EE-TP aim to provide a sustained reduction or elimination of plasma thymidine and 2’-deoxyuridine concentrations and thereby relieve the nervous system and muscle of the toxic effects of the accumulated metabolites. EE-TP has the pharmacological advantages of prolonging the circulatory half-life of the enzyme and potentially minimising immunogenic reactions, which are frequently observed in enzyme replacement therapies administered by the conventional route^[48,77,84,97]^.

Clinical experience with EE-TP is limited to the compassionate use in five individual cases with participants receiving treatment in accordance with the provisions of Schedule 1 of The Medicines for Human Use (Marketing Authorisations Etc.) Regulations SI 1994/3144, where Schedule 1 provides an exemption from the need for a marketing authorisation for a relevant medicinal product, which is supplied on an individual patient basis to fulfil a “special need”. In the early stage of development, the encapsulation of thymidine phosphorylase was performed using a manual process, but for the clinical trial, which has regulatory approval in the United Kingdom, an automated encapsulation process will be employed^[98]^.

In the first proof of concept study, the administration of one dose of EE-TP (34 U/kg body weight) was reported to decrease the urinary excretion of thymidine and 2’-deoxyuridine at three days post infusion to 6% and 13%, respectively, of the amounts excreted pre-therapy. Plasma concentrations of these metabolites were shown to decrease in parallel^[48]^. Escalation of the EE-TP dose in the treatment of four further patients (from 3.7 to 108 U/kg body weight/4 weeks) was reported to reduce or eliminate plasma and urine thymidine and 2’-deoxyuridine levels. Three months after initiating therapy, one patient gained 4 kg in weight, coinciding with a decrease in nausea and vomiting, increased ability to walk longer distances and an increase in the physical and mental components of the SF_36_ health and well-being survey. In another patient, significant clinical improvements were reported in bilateral muscle power (wrist and first finger abduction, knee flexion, ankle dorsiflexion and great toe extension), gait and balance, sensory ataxia and fine finger function, after 23 months of monthly EE-TP administrations. Body weight increased from 57.4 to 63.2 kg, and a fall in plasma creatine kinase activity from 1200 U/L pre-therapy to levels within the normal reference range was reported^[77,84]^. Patient reported outcomes included an ability to walk longer distances, climb stairs without assistance, tie shoe laces, feel the sensation of sand and a return to weight training and guitar playing^[84]^.

No safety issues were reported other than mild reactions to EE-TP infusions in two patients. These were recorded as being transient, occurring within the first 5-10 min of EE-TP infusion and could be managed by pre-medication with antihistamine and corticosteroid anti-inflammatory drugs^[77,84]^. The development of specific anti-thymidine phosphorylase antibodies were reported in only one patient^[77,99]^.

#### AHSCT

AHSCT is one of two therapeutic approaches under investigation which offers the potential of a permanent normalisation of plasma metabolites and restoration of thymidine phosphorylase activity^[82,83,94,100]^. In 2015, Halter *et al*.^[100]^ conducted an international retrospective study of 24 patients with MNGIE who received AHSCT from related or unrelated donors between 2005 and 2015. Of these 24 patients, 15 patients had died, nine from transplant-related mortality and six from their disease. The remaining nine patients were alive 283-2535 days post-transplant and showed that the plasma metabolite concentrations decreased to nearly undetectable levels. Clinical improvements after AHSCT were reported in these cases and others patients subsequently transplanted, including increased body weight and improvements in hearing, swallowing, muscle strength, gastrointestinal manifestations and peripheral neuropathy^[82,83,94,100–102]^ Despite the potential of providing a cure, AHSCT is limited by the availability of a matched donor and the poor clinical condition of patients. The procedure requires aggressive conditioning and immunosuppressive chemotherapy, which is poorly tolerated by sick patients. The procedure carries a mortality risk of 63%, with survival being dependent on an human leukocyte antigen 10/10 match and the absence of gastrointestinal and liver disease^[46,83]^. Gastrointestinal disease is a critical factor in the pathophysiology of graft versus host disease and can lead to the activation of host antigen presenting cells and donor T-cells^[103]^. Patients with MNGIE are likely to present a higher risk of developing gut graft versus host disease because of the disease-related gastrointestinal damage. Disease-related hepatopathies, including hepatic steatosis, hepatomegaly, increased transaminases, abnormal liver function, triglyceride hyperlipidaemia and cirrhosis, have been reported in MNGIE and thus the presence of liver disease should be established prior to transplantation^[16,79]^. The use of parental nutrition and the long-term effect this is likely to have on the liver should also be considered. A published consensus proposal for standardising the AHSCT procedure in patients with MNGIE recommends that patient recruitment should be restricted to those having an optimal donor and without irreversible end stage MNGIE disease^[46,100]^. The potentially risky procedure presents a dilemma for patients who are oligosymptomatic.

#### OLT

OLT is the second treatment approach offering the potential of a permanent normalisation or reduction of plasma metabolites and restoration of thymidine phosphorylase activity^[85,104]^. The rationale is based on the high expression of thymidine phosphorylase in hepatic tissues of unaffected individuals, this being six times higher than activities found in the bone marrow^[105]^. The procedure was first applied to the treatment of a severely affected patient; 13 months post OLT there were mild improvements in the neurological features, a reduction in the cerebral lactate concentration and a normalisation of the plasma metabolites. No clear changes in gastrointestinal function were recorded and the patient continued to receive parenteral nutrition^[85]^. Two further patients have since received OLT, although it is understood that there are other unreported cases of patients with MNGIE who have been successfully transplanted^[104,106]^. Both patients demonstrated normalisation of metabolites soon after OLT, with one patient having a reported increased body mass index and quality of life 90 days post-transplant and the other patient having an improved walking ability and motor strength, although still requiring total parenteral nutrition at one-year post transplant. Unlike AHSCT, OLT does not require a conditioning treatment. Patients with MNGIE often have disease-related hepatopathies, and, considering that OLT has a high survival rate, this approach presents an ideal solution to simultaneously rescue thymidine phosphorylase activity and replace a diseased liver. However, the administration of parenteral nutritional support post-transplant should be carefully considered due to the association of its use with hepatic steatosis and elevated transaminases^[79]^. OLT is limited by the availability of matched donors and can potentially present risks from transplantation complications and the use of long-term immunosuppression therapy.

### Pre-clinical experimental therapeutic approaches

#### Enzyme replacement with polymeric non-reactors

Another approach to enzyme replacement that has been investigated is the encapsulation of thymidine phosphorylase in polymeric nanoreactors^[107]^. The nanoreactors were designed to have nucleoside specific porin Tsx as channel forming proteins integrated in the walls of the nanoreactor, thus permitting the efficient transport of the enzyme’s substrates and products. *In vitro* studies demonstrated that the nanoreactors were stable, showing no leakage of protein when incubated for four days at 37 °C in mouse serum. However, thymidine phosphorylase activity declined by 50% after three days. The nanoreactors produced no cytotoxic effects on hepatocytes isolated from rats, and no inflammatory responses were observed in mice that received intra-peritoneal administration^[107]^.

#### Gene therapy

Gene therapy offers a potential curative option for MNGIE and two promising gene therapy approaches have been investigated using the *Tymp-/-Upp1-/-* double knockout mouse, a disease model which recapitulates the metabolic imbalances of MNGIE^[108]^. The first approach employed a lentiviral vector which was targeted to the haematopoietic progenitor cells with the aim of restoring the activity of thymidine phosphorylase in the blood cells. Although metabolic correction of the biochemical abnormalities was achieved, the survival time of the treated mice was reduced, which was a consequence of the procedure requiring myelosuppression^[109-111]^. In the second approach, mice were treated with an adeno-associated virus vector containing the *TYMP* coding sequence which was targeted to the liver. These studies revealed a normalisation of the biochemical perturbations without any adverse effects^[112,113]^. These studies support the feasibility of gene therapy for the treatment of MNGIE.

## Conclusion

Patients with MNGIE present with a heterogenous array of symptoms, making diagnosis challenging for non-specialist healthcare professionals unfamiliar with the condition. Patients are invariably underdiagnosed and undergo a number of invasive clinical procedures before obtaining a correct diagnosis by laboratory testing. Several experimental therapies have been developed over the past two decades which have successfully achieved metabolic correction. Clinical responses to treatment have been particularly positive in those patients who are at early stages of the disease trajectory, thus reinforcing the need to achieve a greater disease awareness in the healthcare community and to provide timely access to treatments before the development of irreversible organ damage. With an improved understanding of this disorder, the future is likely to see the identification of alternative therapeutic targets or the application of combined treatment approaches to prevent the specific pathologies of this complex, multisystemic disorder. Untoward effects of thymidine phosphorylase overexpression and deoxyribonucleotide pool modifications will require careful surveillance.
